# Allocentric Spatial Memory Performance in a Virtual Reality-Based Task is Conditioned by Visuospatial Working Memory Capacity

**DOI:** 10.3390/brainsci10080552

**Published:** 2020-08-13

**Authors:** Joaquín Castillo Escamilla, José Javier Fernández Castro, Shishir Baliyan, Juan José Ortells-Pareja, Juan José Ortells Rodríguez, José Manuel Cimadevilla

**Affiliations:** 1 Department of Psychology, University of Almeria, Carretera de Sacramento s/n, C.P. 04120 Almeria, Spain; adx94@yahoo.es (J.C.E.); castro_ual@hotmail.com (J.J.F.C.); jjortells@hotmail.com (J.J.O.-P.); jortells@ual.es (J.J.O.R.); 2 Health Research Center, University of Almeria, Carretera de Sacramento s/n, C.P. 04120 Almeria, Spain; 3 Department of Psychobiology, UNED, Calle Juan del Rosal, 10, C.P. 28040 Madrid, Spain; shishirbaliyan@gmail.com

**Keywords:** spatial orientation, navigation, spatial learning, hippocampus, executive functions

## Abstract

Traditionally, the medial temporal lobe has been considered a key brain region for spatial memory. Nevertheless, executive functions, such as working memory, also play an important role in complex behaviors, such as spatial navigation. Thus, the main goal of this study is to clarify the relationship between working memory capacity and spatial memory performance. Spatial memory was assessed using a virtual reality-based procedure, the Boxes Room task, and the visual working memory with the computer-based Change Localization Task. One hundred and twenty-three (*n* = 123) participants took part in this study. Analysis of Covariance (ANCOVA) revealed a statistically significant relationship between working memory capacity and spatial abilities. Thereafter, two subgroups *n* = 60, were formed according to their performance in the working memory task (1st and 4th quartiles, *n* = 30 each). Results demonstrate that participants with high working memory capacity committed fewer mistakes in the spatial task compared to the low working memory capacity group. Both groups improved their performance through repeated trials of the spatial task, thus showing that they could learn spatial layouts independent of their working memory capacity. In conclusion, these findings support that spatial memory performance is directly related to working memory skills. This could be relevant for spatial memory assessment in brain lesioned patients.

## 1. Introduction

Spatial memory is a basic ability used to properly orientate ourselves in our environmental surroundings. It involves a complex network, affecting frontal, and parietal and occipital areas [1]. Specifically, the hippocampal system is one of the most important structures in this network [2,3,4] and a malfunction in this system can lead to inefficient cognitive mapping, navigational problems, or topographical disorientation, especially when using allocentric spatial representations based on the knowledge about spatial stimuli available in the environment [5,6].

In recent years, different studies have further explored the frontotemporal network’s central role in spatial memory performance [7,8,9]. This frontal function allows us to actively manipulate information for executing complex tasks [10] and favors the maintenance of relevant information while discarding that regarded as irrelevant [11]. Both regions, medial-temporal and frontal, are directly and indirectly connected [12]. Thus, temporal lobe dynamics are suggested to modulate prefrontal physiology and functions. Common networks between working memory (WM) and spatial memory circuits are involved in a delayed match-to sample WM task [13]. This relationship is bidirectional, since it is generally accepted that deficits affecting the temporal lobe function, such as Alzheimer’s disease or schizophrenia, influence performance in WM tasks, resulting in lower scores [14,15]. Conversely, a deficit in working memory capabilities (WMC) could cause spatial alterations, preventing the execution of optimal search strategies [16].

It has also been consistently reported that spatial memory span varies significantly across individuals [17], which is also applicable to precision in spatial memory representation [18]. Searching for reliable methods to determine the spatial working memory span in each individual, the Change Localization Task [19] was used, wherein subjects had to detect the color change in one of four circles presented. Performance in this task seems to provide an accurate measure of the amount of information available in the short-term storage system [19] and it has shown strong correlations with different measures of higher cognitive abilities [19,20,21].

Furthermore, spatial memory assessment has significantly expanded with the development of virtual reality-based tasks. These tests have proven to be very sensitive to behavioral disturbances and have been successfully applied to different samples [22,23,24,25,26,27]. An example of this paradigm is the Boxes Room task [28], a virtual reality active navigation task based on allocentric spatial cues. One of its main advantages is the feasibility to be adapted into a variety of difficulty levels, which, alongside free navigation, further contributes to disclose differences in spatial memory when compared to traditional tasks [27,29].

Accordingly, the main goal of our study was to explore the potential relationship between working memory capacity and spatial memory performance in an allocentric spatial memory task. Hence, hypothesizing that achievements in one domain would affect another, participants were compared in the Boxes Room task, taking into account their WM capacity in the Change Localization Task. Following previous evidences [18,30], we hypothesized that participants with a higher WM capacity should obtain better scores in the spatial task.

## 2. Materials and Methods

### 2.1. Participants

One hundred and six women (*n* = 106; $\bar{X}$ age = 21.33; *SD* = 4.82) and seventeen men (*n* = 17; $\bar{X}$ age = 19.41; *SD* = 2.37), all of them undergraduate students from the University of Almeria, were recruited to participate in the study. They had normal or corrected vision at the moment of the assessment. Some exclusion variables were considered: diagnose of psychological or psychiatry disorders, drug consumption, traumatisms, or any other condition that could interfere with the performance. The participants were informed in advance about the aims and procedures of the experiment. All participants gave written consent and were informed that they were free to leave the experiment at any time. The study was approved by the Ethical Committee of the University of Almeria and conducted in accordance with the Board of the European Community 2001/20/EC and the Helsinki Declaration for Biomedical Research Involving Human Beings. Research hypotheses were not revealed to participants.

### 2.2. Procedure

All participants were tested individually and received verbal and written instructions before each test. Tests were administered in the following order: interview, Change Localization Task, and The Boxes Room task. The full procedure lasted about 30 min.

- Working memory capacity

**Figure 1:**
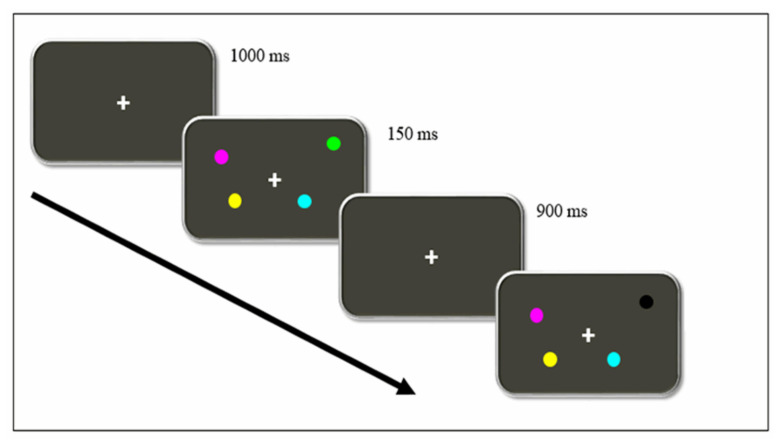
Timeline of events of a single trial in the Change Localization Task. Participants must identify which circle changed its colors between each exposure. In this example, they must point the mouse to the black circle.

The Change Localization Task [19,20,21] was used to assess working memory capacity (WMC). The task was designed using the *ePrime 2.0* software (Psychology Software Tools) and executed in a portable computer. Viewing distance was approximately 60 cm. Figure 1 represents the sequence of events presented in each trial.

Firstly, a fixation point was presented for 1000 ms on the screen. After that, four colored circles were shown for 150 ms. Minimal radius from the fixation point to the closest stimulus was 3,36º, and 6,24º for the farthest. All four circles were differently colored (orange, green yellow, cyan, magenta, blue, red, white, and black). Afterward, the initial black screen was shown again for 900 ms, followed by another set of four circles, whose colors and positions were the same as those in the previous set except for one, which was colored differently. The participants were to choose, using the laptop mouse, which of the four circles had changed their color.

An initial practice block, consisting of 12 trials, was executed in order to familiarize participants with the flow of the task and instructions. Feedback of their performance was given to participants. After a while, participants performed two consecutive experimental blocks of 32 trials per block, with an interval break between the experimental blocks. The task lasted about 8 to 10 min, and the total number of correct responses and errors were registered.

- Spatial memory

The Boxes Room [28] is a virtual spatial memory task consisting in a room with 16 brown boxes in a 4 × 4 disposition. Inside the room, there are several stimuli that disambiguate spatial locations, including a door, a window, and several pictures. Participants can navigate through the room to reach different boxes by means of a joystick.

The goal is to find the position of 5 rewarded boxes located always in the same place during the experiment. A box can be opened by hovering the cursor over it and pressing the joystick button. If the box was rewarded, it changed its color from brown to green and a pleasant melody sounded. In contrast, if the box opened was incorrect, it turned to red color followed by an unpleasant melody sound. The four cardinal points were used as starting positions changing semi-randomly, avoiding egocentric solutions of the task. Participants were not informed about spatial strategies or the position of the reward boxes.

**Figure 2:**
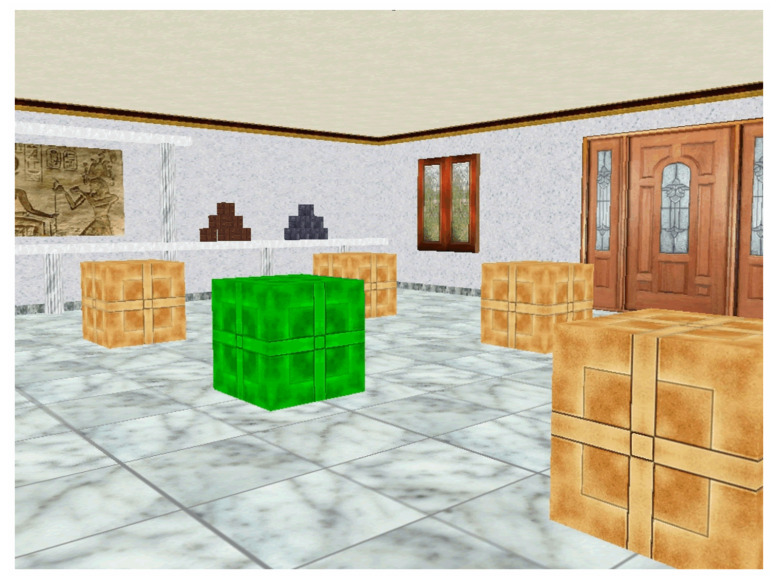
The Boxes Room Task. Participants explore the room using a joystick. The rewarded boxes turn green when located, whereas non rewarded boxes turn red. Unopen boxes remain brown. Several stimuli help to disambiguate spatial locations, such as a door, a window, and pictures hanging on the walls.

Before starting, participants were given written instructions of the general procedure of the task, and indications on how to use the joystick to move and open boxes inside the virtual room. The task consisted of 10 trials. The trial ended when all rewarded boxes were found or when 150 s had elapsed. The number of errors were registered. A layout of the experimental environment is presented in the Figure 2.

### 2.3. Statistical Procedure

Before the proper data analysis, the K-index for each participant in the Change Localization Task was calculated. The proportion of correct responses from each participant was multiplied by four in order to calculate their visual working memory capacity represented by a K-index, based on the Pachsler/Cowan equation [31]. It is designed to identify the number of items present in WM based on hit and false alarm rates. The proportion of correct responses was multiplied by four (equaling the number of stimuli) to obtain the K-Index as the WMC reference. This equals the mean number of colored circles memorized by a participant. Therefore, the scores ranged from 0 to 4 (see [21]). All participants were above chance level for the task.

Trials in the Boxes Room task were grouped in three blocks to reduce data dispersion (Block 1 = trials 2–4; Block 2 = trials 5–7; Block 3 = trials 8–10). The first trial was performed randomly, since participants did not initially know the location of the rewarded boxes. Thus, the first trial was discarded from subsequent analyses to avoid its interference.

A repeated measures ANOVA (Gender × Block) was run to determine if there is a relationship between spatial memory and gender. Thereafter, in order to know whether participants’ performance in the spatial memory task was modulated by individual differences in WMC, we conducted a further analysis of covariance (ANCOVA) treating trial Blocks in the spatial memory task as a within-subjects factor, and WMC (K scores) as a continuous covariate variable. For similar analyses see [21,32,33].

In order to classify participants based on their WMC for comparisons in their spatial memory performance, we used an extreme-groups design approach [34,35]. Therefore, participants who scored, respectively, in the upper (*K* > 3.3) and lower (*K* < 2.88) quartiles in the Change Localization task of our overall sample, formed the “High-WMC” and “Low-WMC” groups, respectively. A student T test was applied to check whether differences between the WMC groups were statistically significant and, thus, effectively represented different WMC levels.

Finally, a further, more specific ANOVA with those WMC subgroups was run, treating the Block of trials (three blocks) as a within-participants factor, and WMC (High vs. Low groups) as a between-subjects variable. Statistical analyses were run using the program *IBM SPSS* (Version 22) with a significance level of *p* < 0.05.

## 3. Results

### 3.1. Comparisons Regarding Gender

A repeated measures ANOVA was applied to the number of errors in the spatial memory task (Gender—male or female × Block of trials) with repeated measures in the last variable and with *K* index as a co-variable. Normality criteria were not met by any Block × Gender combination with the Shapiro–Wilk procedure (*p* < 0.050). The Box’s Test for Equivalence of Covariance Matrices was not statistically significant (*p* = 0.612), supporting the null hypothesis of covariance matrixes equality. The Mauchly Sphericity Test was not fulfilled for Block (χ2 (2) = 0.82, *p* = 0.000). Considering this, we chose multivariate statistics for the analyses. There was no effect of Block (F(2,119) = 2.30; *p* = 0.105), or Gender (F(1,20) = 0.84; *p* = 0.361) or the interaction of Gender x Block (F(2,119) = 0.137; *p* = 0.872). There was a main effect of the K co-variation (F(1,120) = 5.68; *p* = 0.019). Thus, all participants (*n* = 123) were considered in the subsequent analyses regardless of their gender.

**Figure 3:**
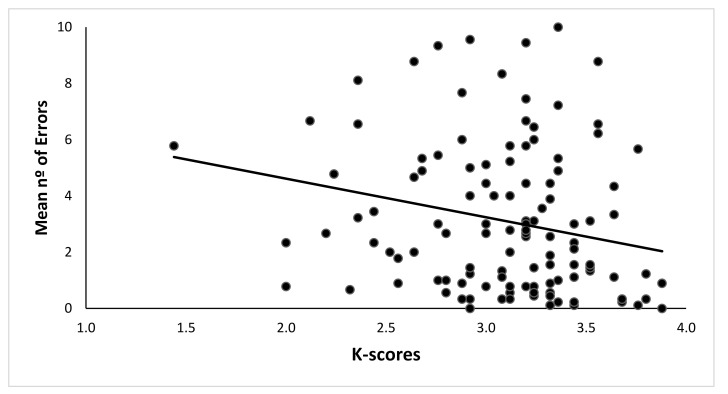
Dispersion of mean number of errors in the Spatial Memory Task due to the K-index. High K indexes are correlated with better performance, and thus, a smaller number of errors were made.

A Pearson correlation between the K-index in the change localization task and the mean number of errors in the Boxes Room Task was estimated. A negative significant correlation between the K-index and the mean number of errors was found (*r* = −0.221, *p* = 0.014). Hence, a higher WMC would be associated with fewer errors in the spatial memory task. See Table 1 for the complete correlation chart for each block of trials, and Figure 3 for data dispersion.

### 3.2. WMC and Spatial Memory Performance

**Figure 4:**
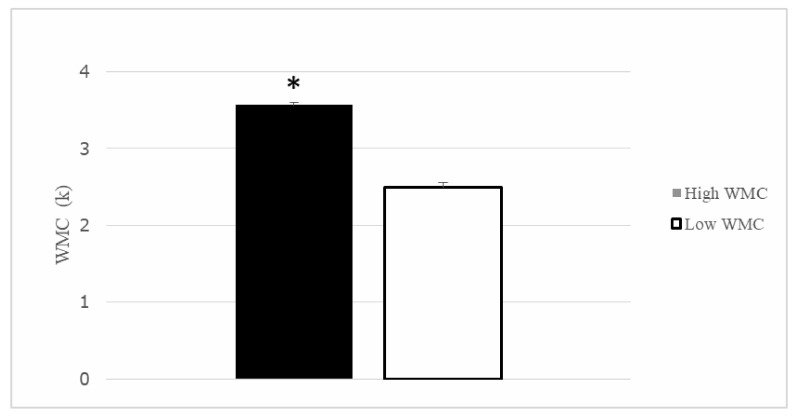
Working Memory Capacity (K index) for the Higher and Lower working memory capabilities (WMC) participants. Groups differed in their WMC. Mean + SEM. * *p* < 0.05.

After the division in quartiles for the K-index, thirty participants were included in the Q1 (>3.36), composing the High WMC group ($\bar{X}$ = 3.57, *SD* = 0.155), and the other thirty participants formed the Q4 (<2.88) or low WMC group ($\bar{X}$ = 2.50, *SD* = 0.321). Both groups differed in their performance in the Change Localization task (*t* (58) = 16.43, *p* < 0.001) (see Figure 4). The remaining participants from Q2 and Q3 were not considered in the subsequent analysis.

**Figure 5:**
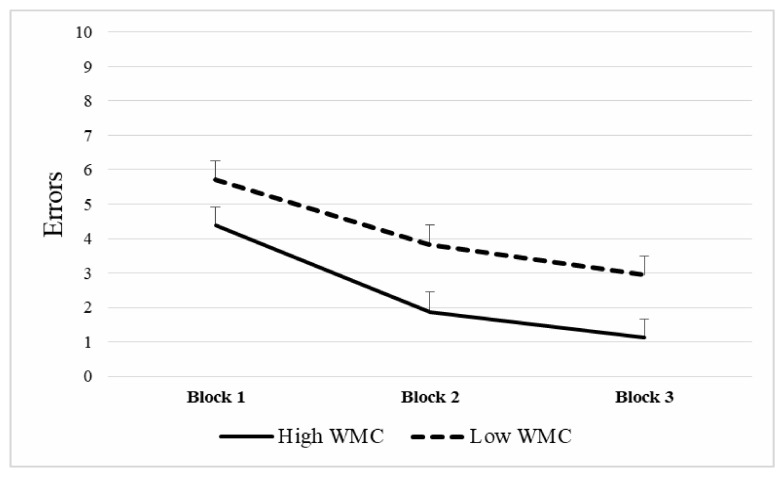
Mean number of errors in The Boxes Room task according to working memory capacity (WMC). Note that higher WMC was related to better performance in the task through the different blocks of trials. Mean + SEM.

A repeated measures ANOVA was applied to the number of errors in the spatial memory task, (Group—high **vs.** low WMC- × Block of trials), with repeated measures in the last variable. Normality assumption through Shapiro–Wilk procedure was met only for the low WMC group in the first block of trials (*p* = 0.400) but not of the rest of Block × WMC combinations (*p* < 0.050). Box’s Test for Equivalence of Covariance Matrices was not statistically significant (*p* = 0.130), which supports the null hypothesis of covariance matrixes equality. The Mauchly Sphericity Test was not fulfilled for Block (*χ2* (2) = 0.819, *p* = 0.003). Thus, results were interpreted using multivariate statistics. There was a significant main effect in Group (*F*(1,58) = 5.73, *p* = 0.020, *η2* = 0.090), such that Low-WMC participants showed a higher error rate in the spatial memory task ($\bar{X}$= 4.15) than High-WMC participants ($\bar{X}$ = 2.45). The main effect of the Block of trials was also significant (*F*(2,57) = 39.64, p < 0.0001, *η2* = 0.582). Post-hoc analyses using the Bonferroni procedure revealed that participants committed more errors in the first block ($\bar{X}$ = 5.05; *SD* = 3.04), when compared to the second block ($\bar{X}$= 2.83; *SD* = 3.32; *p* = 0.00) and the third block ($\bar{X}$ = 2.03; *SD* = 3.10; *p* = 0.00). Second and third block error scores also differed (*p* = 0.006), with more errors in the former compared to the latter (see Figure 5).

## 4. Discussion

The main goal of this study was to determine the relationship between visual working memory capacity (WMC) and performance in an allocentric spatial memory task. Our study demonstrated that participants with a higher visual WM performance committed fewer errors than those with a lower WMC, thus confirming the tendency found in the initial ANCOVA and correlational analyses. Accordingly, visual WM skills are related to the functionality of neural circuits involved in spatial memory performance in an active navigation task.

To determine WMC abilities, we used the Change Localization Task [19,20,21], which measures visual working memory performance. It is worth noting that this simple task is a pure index of our short-term buffer [19]. The measures obtained by changing detection/localization tasks have shown a strong relationship with other higher cognition measures such as attention control, maintenance, and retrieval of different types of memories [20,36]. The Change Localization Task has no time limit and requires a reduced number of trials to obtain reliable results, reducing fatigue, which could produce an impact on performance [37]. The guessing effect is also reduced because its chance level is 25%. In addition, the type of stimuli used do not allow the use of verbal coding strategies [21]. Using colored circles also favors precision in performance [38]. In our study, two groups were formed with those scoring in Q1 and Q4. Groups differed between themselves in this domain, and thus, represented differences in WM capacity. It is important to consider that the extreme-groups methodology for dividing participants in their WMC have some limitations for extrapolations in the general population [31].

Moreover, spatial memory performance was assessed with the Boxes Room Task [28]. This is a virtual reality-based task developed in our laboratory and applied to several populations during the last ten years [25,29,39,40], showing a good sensitivity to discriminate between groups in a variety of domains [27,41,42].

In our study, WM capacities of participants were related to a differential number of errors in the Boxes Room task, with a significant negative correlation between the K-index and the mean number of errors (total and per block). Furthermore, a main effect of Group in the repeated measures ANOVA was found. Thus, high WMC participants outperformed those with lower WMC. It is also worthy to note that both groups (high WMC and low WMC) improved their performance as stated by the significant main effect of Block, since the number of errors decreased with training after a few trials, as the first block of trials (2–4) showed a higher number of errors compared to the second (5–7) and third (8–10), which also differed between them. According to this, 10 trials were more than enough to learn the task regardless of WMC. This tendency states that the learning process is different through trials. Thus, in the initial trials, it is necessary to learn the context and relationships between the cues available and the rewarded positions, as expected from the allocentric strategy [5]. It is at this early stage when the spatial relationships between the different cues are encoded, enhancing the cognitive demands, and impacting performance. Once spatial information is acquired, the task becomes less demanding and more automatic, as participants become familiar with stimuli and procedure, resulting in fewer errors [43]. Thus, the WMC is effectively indicating the ability of our participants to maintain and manage information required for accurate orientation. It is necessary to highlight that, as suggested by previous works [44], a better WMC could be required for transforming spatial cues into stable representations and keeping track of them. Furthermore, this process is essential in this spatial memory task, since egocentric solutions are avoided by changing semi-randomly the starting point. Hence, participants had to determine their position in the room to effectively locate the rewarded positions.

Motivational or attentional processes could account for group differences. However, different factors should be considered. The whole experiment lasted about 30 min. Accordingly, young students had no time to get fatigued. The score of the low WMC group in the Change Localization task indicates that their performance was clearly over the chance level (see Figure 3). In addition, both groups improved in the spatial task reducing the number of errors to levels incompatible with the lack of motivation or attention.

Our results suggest that frontoparietal networks involved in visual working-memory are directly related to spatial memory abilities that depend on a wide network, including the hippocampal system, as supported by previous evidence. Hence, the medial prefrontal cortex has a role in the retrieval of remote spatial memories [45]. Precisely, the ventromedial prefrontal cortex would be implicated in integrating information represented in the hippocampus, and, subsequently, would suppress irrelevant information based on these integrations. Notice that both structures are involved in working memory processes and that they show activation in working memory demanding tasks in humans [46]. This interaction is explained by the direct and indirect connections [12] and the theta band connection between both structures [46,47].

In addition, it was reported that prefrontal lesions modified the activity of hippocampal place cells, reducing the mean firing rate and stability of the firing fields across time [48]. This could affect behavior, since prefrontal cortex underperformance would increase noise and irrelevant information by disinhibiting the control of incoming signals entering the hippocampus [49]. Other authors proposed a model of distributed spatial cognition system: the hippocampal system would provide redundant spatial representations required for navigation and the prefrontal cortex would elaborate a more complex representation, including emotional, motivational, and reward-dependent information [50]. Thus, the prefrontal cortex damage would be related to failures in topological representation and action selection.

Some other studies have addressed the relationship between frontal and temporal regions by disconnecting hippocampal and prefrontal areas in rats. These works demonstrated that bilateral lesions of either prefrontal cortex or hippocampus can cause a memory deficit and topographical disorientation [51,52].

Furthermore, many other areas contribute to visual working memory capacity such as the visual and parietal cortices or even subcortical structures (for a review see [53]). It would be simplistic to reduce the influence on the prefrontal cortex.

Finally, it is important to consider that spatial memory has been generally considered sexually dimorphic [22,54,55,56]. However, no differences were found in our study due to gender, contradicting these claims. It should be noted that sexual dimorphism could also be modulated by familiarity and time of exposition to stimuli [57], difficulty level [58], or age [59], explaining the lack of differences. An important limitation of this study is the unbalanced number of men and women included. Due to a limitation in recruiting men as a result of a limited availability, the proportion between both genders is highly favorable to women, which suggests being cautious about the extrapolation of our data in this domain.

## 5. Conclusions

Our results provide additional behavioral evidence to prefrontal cortex-temporal lobe relationship. Inter-individual differences in visual WM modulates the spatial memory performance, supporting previous functional and anatomical findings in favor of a neural link between the mentioned brain regions. This is especially relevant in neuropsychological studies with brain lesioned patients since they suggest that other cognitive functions could account for spatial memory performance.

## Figures and Tables

**Table 1:** Correlation between the K-Index from the Change Location Task and Trial Blocks in the Boxes Room Task.

	Block 1 (Trials 2–4)	Block 2 (Trials 5–7)	Block 3 (Trials 8–10)
**K-Index**	−0.176	−0.237	−0.180
**Significance**	0.051	0.008	0.046
